# A new evaluation method for +Gz tolerance with loratadine by using a near-infrared spectroscopy

**DOI:** 10.1186/1476-5918-7-3

**Published:** 2008-01-28

**Authors:** Akihiko Onozawa, Azusa Kikukawa, Yoshinori Miyamoto

**Affiliations:** 1 Aeromedical Laboratory, Japan Air Self-Defense Force, Tachikawa, Tokyo, Japan

## Abstract

**Background:**

Loratadine (Claritin^®^), an over the counter antihistamine in U.S. and UK, is acceptable for use without adverse side effects by aircrew with mild or moderate allergic or other situations requiring an antihistamine. Although +Gz (head to foot direction) tolerance testing for aircrew with loratadine has not been documented in the published literature, it is commonly accepted that loratadine dose not effect +Gz tolerance. The purpose of this study was to offer and validate a new evaluation method for +Gz tolerance testing with loratadine by using a near-infrared spectroscopy (NIRS).

**Methods:**

A double-blind, placebo-controlled, randomized, crossover protocol was used to administer 10 mg of loratadine or placebo in nine healthy subjects. The subjects didn't wear anti-G suit. The +Gz exposure profiles consisted of, in series, a gradual onset ran (0.1 G·sec^-1^) to the subject's visual end-point (peripheral light loss) or loss of consciousness (GLOC), and rapid onset run (1.0 G·sec^-1^) to the subject's same end-point. In this study, G-level tolerance was defined as the +Gz level at visual end-point and/or at GLOC. As a subject's G-duration tolerance, we measured the total time (seconds) during rapid onset run. Otherwise, to confirm the effect of loratadine on +Gz tolerance, we measured the cerebral NIRS variables (hemoglobin concentration changes and tissue oxygenation index) as a new quantitative method for +Gz tolerance during a centrifuge experiments.

**Results:**

No significant differences were observed in +Gz tolerance (+Gz level, duration time and NIRS variables) between subjects taking loratadine and placebo.

**Conclusion:**

Our results demonstrate that loratadine has no detectable effect on +Gz tolerance by using a new method with cerebral NIRS variables and the traditional method with +Gz level and duration time. This study represents the first use of a quantitative parameter such as cerebral NIRS variables to assess the effects of a drug on acceleration tolerance.

## Background

Older first-generation antihistamines, such as diphenhydramine and chlorpheniramine, are unsuitable for use by aircrew during flight duties due to their well-documented sedating side effects. Loratadine (Claritin^®^) is a newer, second-generation antihistamine available over-the-counter in the US and UK. It has been declared by various civil and military authorities to be acceptable for use by aircrew with mild or moderate allergic symptoms or other situations requiring an antihistamine (e.g. urticaria) due to its well documented lack of adverse side-effects, including sedation. Previously, it has been proposed that a single 10 mg dose of loratadine will not effect flying performance [1]. A single 10 mg dose had no effect on airline pilot performance in flight simulators [2]. Moreover, ingesting 10 mg of loratadine daily did not demonstrate sedative effects or other impairments of cognitive motor performance [3]. Recently, a single 5 mg dose of desloratadine (an active metabolite of loratadine) did not cause sleepiness nor did it impair the performance of tasks associated with flying ability [4]. Accordingly, loratadine is advantageous to aircrew suffering from allergy symptoms due to its lack of sedation or other central nervous system (CNS) effects commonly associated with older forms of antihistamine therapy. For high-performance aircraft pilots, even though no CNS effects have been demonstrated, loratadine should be use cautiously and close scrutiny, but no flying restrictions are necessary provided there are no side-effects including for ability of +Gz tolerance.

It is commonly accepted loratadine dose not affect +Gz (head to foot direction) tolerance, although + Gz tolerance testing with loratadine that has not been documented in the published literature [3]. In fact, the air forces of several nations have treated pilots of high-performance aircraft with loratadine for a decade already. In general, high-performance aircraft, e.g., fighters and trainers need an anti-G straining maneuver and/or +Gz protection in the military +Gz environment. Consequently, their ability to tolerate the +Gz acceleration is of major concern to the military operational communities and a challenge to acceleration physiologist [5]. More recently, as mentioned earlier, loratadine is available in several countries without a prescription. It is more difficult for people including medical authority to understand the effects of self-medication loratadine than prescription loratadine. Accordingly, a better understanding of the effects of loratadine with published literature medically, pharmacologically, acceleration physiologically and flight safely, is important.

In a controlled experimental setting, +Gz tolerance is examined using a model of +Gz stress (commonly specific runs in a centrifuge). Centrifuge profiles used in examining +Gz tolerance may assess +Gz intensity (e.g., peak +Gz level) or +Gz duration [6]. Thus in assessing relaxed acceleration tolerance, these include the assessment of peripheral light loss (PLL) or central light loss using a display system/lights, or the use of 60° peripheral visual loss as an end point with subject self-report. Other methods of assessing have also used ear opacity [7], and blood pressure [8,9]. Basically, the ability to tolerate levels of +Gz exposure is primary a function of adequate blood flow to the brain [5].

Near-infrared spectroscopy (NIRS) is a non-invasive method of quantifying changes in cerebral hemodynamics from changes in the absorption of near-infrared light by oxyhemoglobin (O_2_Hb) and deoxyhemoglobin (HHb), and the theory of NIRS is described in detail elsewhere [10-14]. The recently developed NIR spatially resolved spectroscopy has been confirmed to provide frontal cortical tissue hemoglobin saturation [tissue oxygenation index (TOI)] [15,16]. The main use of these techniques in clinical, biomedical and aeromedical research has focused on the strong signals available from the hemoglobin chromophores and their relation to cerebral physiology and pathophysiology [17]. Recently, NIRS is increasingly used for cerebral hemodynamics and oxygenation changes, and it's a relatively new technique which allows non-invasive measuring changes in oxygenated hemoglobin concentration [12,18,19], including first recorded in a laboratory centrifuge [20,21], saturation of cerebral vascular space measurements [22], in-flight cerebral oxygen status [23,24], a study of the almost loss of consciousness [25], consciousness monitoring during high +Gz exposure [26], computer modeling of acceleration effects [27] and electroencephalographic correlation study concerning Gz-induced loss of consciousness [28].

The purpose of this study was to offer and validate a new method for +Gz tolerance testing with loratadine by using a NIRS. Thus, to confirm the effect of loratadine on +Gz tolerance in healthy subjects during a centrifuge experiments, we measured the cerebral NIRS variables [HHb, O_2_Hb, cHb (HHb + O_2_Hb) and TOI] changes. In addition, to compare the effect of loratadine on +Gz tolerance, we also measured +Gz-level tolerance and +Gz-duration tolerance as a traditional method [5].

## Methods

A double-blind, placebo-controlled, randomized, crossover protocol was used to administer 10 mg of loratadine (a powdered tablet) capsule or placebo (lactose) capsule in 9 volunteer subjects (8 males and 1 female). The mean age of the 9 subjects was 34.3 years (age 34.3 ± 6.5 yr, mean ± SD). All subjects were in good health and were experienced centrifuge riders. None was on any medication. The intake of drink containing caffeine and smoking were prohibited from rising in the morning to the end of the test. The study protocol was approved by the Ethical Committee of Aeromedical Laboratory, Japan Air Self-Defense Force (JASDF). All subjects were fully briefed on the scope of the experiment, and informed consent was obtained from them before experiment. Each subject's experiment was measured on two separate days in the same order, at similar time points, and under the same conditions. Each day the subject took a loratadine capsule or a matched placebo capsule 90 minutes prior to the centrifuge experiments. On the experiment days, the administration of medication took place under the supervision of a member of our laboratory staff to assure mode and time of proper drug administration. The subjects were given two trials of the centrifuge exposure, each trial separated by a two-week interval. This was to prevent any effects of repeated trials over short time intervals and to ensure a complete drug washout had taken place.

The subjects were seated on the human centrifuge at the JASDF Aeromedical Laboratory Iruma (8-m radius, Shimadzu, Kyoto, Japan). The seat had a seat-back to seat-pan angle of 90° and a seat-back angle of 13° with respect to the vertical.

**Figure 1:**
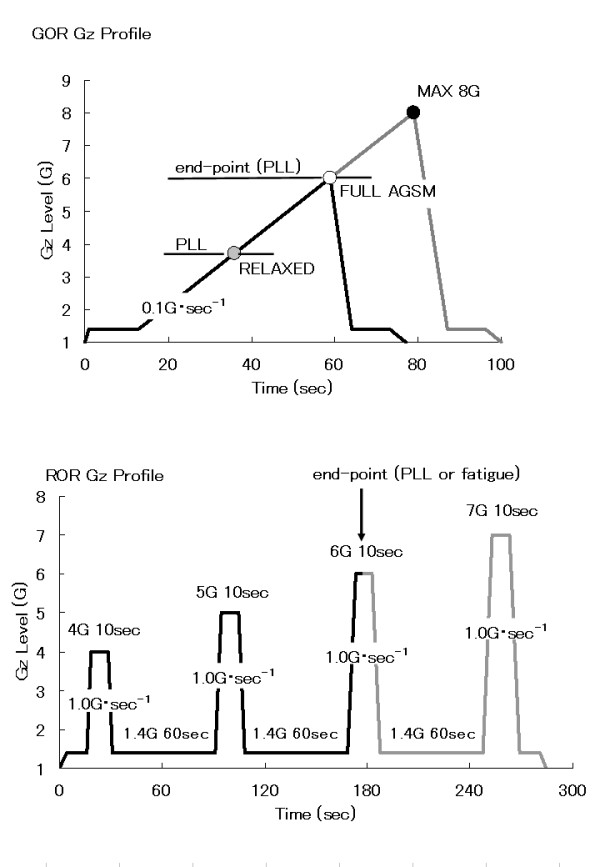
**Gz exposure profile**. GOR: gradual onset run, ROR: rapid onset run, PLL: peripheral light loss, AGSM: anti-G straining maneuver.

The +Gz exposure profiles consisted of, in series, a gradual onset run (GOR, 0.1 G·sec^-1^) to the subject's visual end-point or loss of consciousness (GLOC), and rapid onset run (ROR, 1.0 G·sec^-1^) to the subject's same end-point. The end point was reported by subjects at PLL. A subject was said to have experienced PLL when the boundary of her or his visual field subtended an angle less than 60°. GOR profile was performed without anti-G suit to determine relaxed tolerance and preceded until the subject's end-point, after which time the subjects began anti-G straining maneuvers. And then 5 min rest at 1.0 G after the GOR, subjects were exposed as a ROR without anti-G suit to a continuous Gz profile of 10 sec of 1.4 G, then 10 sec of 4 G, back to 60 sec of 1.4 G, then 10 sec of 5 G, back to 60 sec of 1.4 G, then 10 sec of 6 G, back to 60 sec of 1.4 G, etc., until the subject reports end-point, GLOC or fatigue (Figure 1). As noted above, the subjects were not provided with G suits, but protected themselves until end-point and/or GLOC by voluntary G protective maneuvers such as muscle straining and L-1 maneuvers.

G-level tolerance was defined as the +Gz level at end-point or at GLOC. Thus we measured relaxed G-level tolerance (which includes involuntary G protection) and straining G-level tolerance (which includes protection afforded by executing the anti-G straining maneuver) during the GOR centrifuge profile. On the other hand, duration time for voluntary fatigue or visual loss has been validated as a reproducible end point which represents an individual's ROR G-duration tolerance. As a duration time, thus we measured the total time (seconds) from the start of ROR at 1.4 G to the subject's end-point above 1.4 G (Figure 1).

Additionally, to confirm the effect of loratadine on +Gz tolerance in healthy subjects during a centrifuge experiments, we measured the cerebral HHb, O_2_Hb, cHb and TOI changes. We used a NIRS system (NIRO-150G; Hamamatsu Photonics K.K., Japan) at three wavelengths (775, 830, and 850 nm). The optical pathlength factor was 5.93 [29]. After cleaning of the subject's forehead with alcohol, the optodes were carefully fixed, using flexible and adhesive fixation pad and double-faced adhesive tape. A light emitter (emitting optode) and a light detector (collecting optode) were placed with a distance of 4 cm between them on the right forehead of the subjects. Each probe was protected from ambient light by surgical tape. Data were collected with sampling intervals of 0.167 sec. A baseline correction was performed about 20 sec before centrifuge experiments. We analyzed the changes in O_2_Hb by subtracting the mean baseline values (before the centrifuge experiment) from the end point values in each +Gz exposure. The mean changes of HHb (ΔHHb), O_2_Hb (ΔO_2_Hb), cHb (ΔcHb) and ΔTOI relative to baseline for each +Gz exposure were calculated for all subjects. The electrocardiogram and heart rate of the subjects were also monitored throughout the experiment. In this study, one of nine subjects experienced GLOC at the ROR profile, the subject was quickly removed from the high +Gz environment. Additional centrifuge run of the subject was not carried out within two weeks.

Wilcoxon singed-rank test were used to determine the significance of the levels of the end-point and NIRS variable changes for the subjects administrated placebo and loratadine during the centrifuge run. Results were expressed as the mean ± standard deviation (SD) at each sampling period for all subjects. A *p*-value of <0.05 was considered statistically significant in all experiments.

## Results

**Figure 2:**
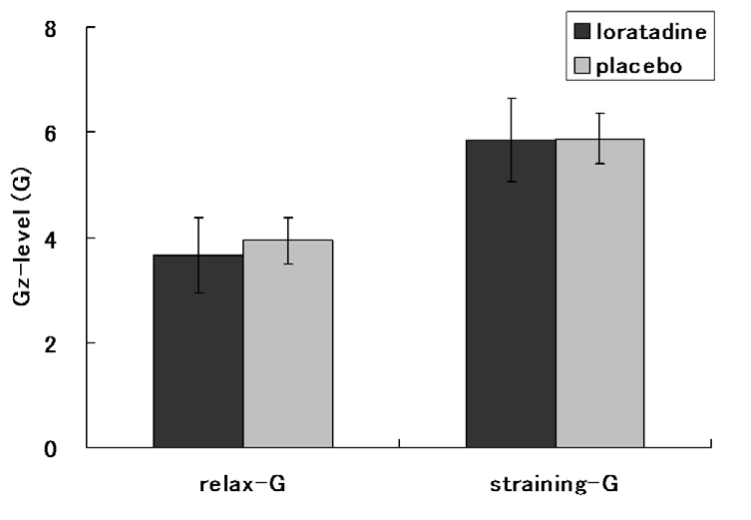
**G-level tolerance on the GOR profile**. Data are presented as mean ± standard deviation, n = 9. GOR: gradual onset run.

**Figure 3:**
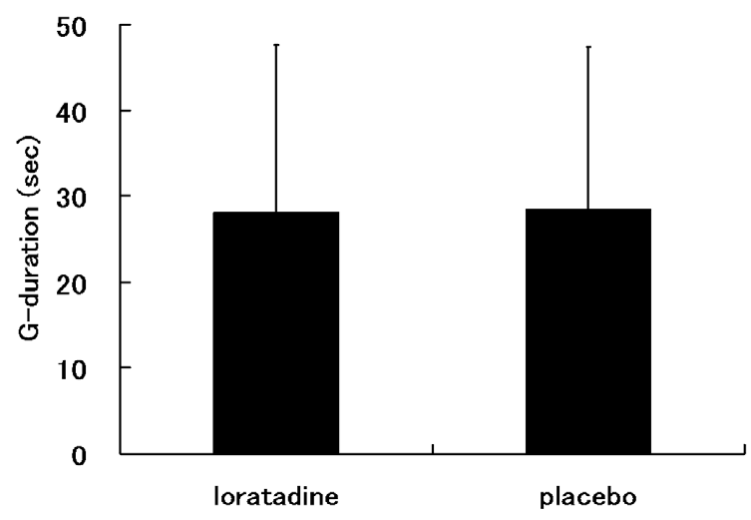
**G-duration tolerance on the ROR profile**. Data are presented as mean ± standard deviation, n = 9. ROR: rapid onset run.

G tolerances are listed as mean (± SD). G-level tolerances during relaxed GOR profile were 3.64 ± 0.72 on loratadine and 3.93 ± 0.43 on placebo (Figure 2). The straining G-level tolerances during GOR profile were 5.86 ± 0.78 on loratadine and 5.88 ± 0.48 on placebo (Figure 2). G-duration tolerances during ROR profile were 28.1 ± 19.5 (sec) on loratadine and 28.4 ± 19.0 (sec) on placebo (Figure 3). NIRS variables at end point between placebo and loratadine during +Gz exposure with GOR and ROR are shown in Table 1. No significant differences observed in all NIRS variables between subjects taking placebo and loratadine during +Gz exposure with GOR and ROR.

**Table 1:** NIRS variables at end point between placebo and loratadine during +Gz exposure with GOR and ROR.

	***GOR***				***ROR***	
		
***NIRS Variable***	***Relaxed***		***Straining***		***Straining***	
			
	***Placebo***	***Loratadine***	***Placebo***	***Loratadine***	***Placebo***	***Loratadine***
***ΔO_2_Hb (μmol·L^-1^)***	-10.5 ± 5.6	-11.1 ± 5.7	-17.4 ± 8.1	-16.9 ± 7.4	-14.7 ± 7.2	-15.0 ± 6.4
***ΔHHb (μmol·L^-1^)***	0.5 ± 2.4	-0.1 ± 3.4	2.8 ± 1.2	1.4 ± 4.6	0.7 ± 3.0	1.9 ± 3.7
***ΔcHb (μmol·L^-1^)***	-11.3 ± 6.8	-12.8 ± 8.6	-13.2 ± 6.8	-15.5 ± 12.6	-13.6 ± 7.0	-12.0 ± 7.6
***TOI%***	67.6 ± 4.5	70.2 ± 6.4	64.8 ± 3.7	67.2 ± 6.9	63.5 ± 4.8	66.3 ± 5.7
***Baseline***	72.4 ± 4.7	73.6 ± 5.1			72.2 ± 4.9	73.7 ± 4.7
***ΔTOI%***	-4.8 ± 2.5	-3.3 ± 3.2	-7.6 ± 2.2	-6.4 ± 4.0	-8.8 ± 1.5	-7.4 ± 3.1

Values are means ± standard deviation. NIRS: near-infrared spectroscopy, GOR: gradual onset run, ROR: rapid onset run, O_2_Hb: oxygenated hemoglobin, HHb: deoxygenated hemoglobin, cHb: O_2_Hb + HHb, TOI: tissue oxygenation index, Δ: change.

## Discussion

Previously, after single oral administration of loratadine (20 mg), mean maximum plasma concentration was found for loratadine at 1.2 hours and for desloratadine (an active metabolite of loratadine) at 1.5 hours, respectively [30]. Then in this study, the subject took loratadine capsule or a matched placebo capsule for 90 min prior to the centrifuge experiments.

As a rule, the term G tolerance was coined to include the magnitude (i.e., level) of the +Gz stressor and the duration of exposure to the +Gz stressor [31]. The traditional method used to determine relaxed G-level tolerance is the symptom loss of vision in a relaxed, upright-seated subject at a specific of G exposure [5]. Otherwise, G-duration tolerance is the length of time in seconds that a subject can continue to maintain vision, usually performing a simple task while exposed to a repeating predetermined G profile that continues until the G exposure is stopped by the subject because of fatigue [5]. In this study, during the GOR centrifuge profile, no significant difference was found between the loratadine and placebo groups in either relaxed or straining G-tolerance (Figure 2). Moreover, no significant difference was observed in G-duration tolerance between the 2 groups as well (Figure 3). As stated above, our results with the traditional method suggest that there were not significant effects of loratadine on tolerance to + Gz acceleration.

In this study, the effect of loratadine on +Gz tolerance was also assessed quantitatively using NIRS. High +Gz exposure is believed to induce cerebral ischemia and perhaps cause a disproportionately greater fall in O_2_Hb tissue levels than those found at 1 Gz [5,24,32,33]. A combination of these effects of G-forces is probably the predominant reasons for peripheral visual field, visual greyout or blackout, and GLOC. Moreover, it is also believed that the ability to tolerate +Gz exposure is primarily a function of adequate blood flow to the brain. Consequently, symptoms that relate to inadequate blood flow to the head are used as a measure of +G-level tolerance [5].

In general, the sum of the concentration of O_2_Hb and HHb (cHb = O_2_Hb + HHb) generally provides a measure of the cerebral blood volume, whereas the individual concentrations of the two forms of hemoglobin are the result of the interplay between physiological parameters such as regional blood volume and blood flow [34]. Otherwise, functional magnetic resonance imaging (fMRI)-NIRS or positron emission tomography-NIRS simultaneous measurement studies have reported that O_2_Hb is strongly correlated with the fMRI signal and regional cerebral blood flow [35-37]. Moreover, a previous in-flight study in an F-15 aircraft, where cerebral oxygen status (O_2_Hb, cHb and TOI) was monitored showed that the effect of +Gz acceleration was to simultaneously lower blood flow in a mirror image fashion to the applied acceleration [24]. This NIRS technology has matured for continuous monitoring of in-flight cerebral oxygen status under various field conditions. However, in-flight study should design preserved flight safety by collecting data on the pilots.

As described above, +Gz exposure is believed to induce cerebral ischemia and perhaps cause a greater fall in cerebral O_2_Hb. Recently, our laboratory study demonstrated the TOI to be a useful variable to evaluate the effect of the anti-G protection system [19]. Accordingly, this accepted hypothesis and based on previous centrifuge experiments including our laboratory study, we believe the measuring of cerebral hemoglobin concentration changes (ΔHHb, ΔO_2_Hb and ΔcHb) and TOI changes during +Gz exposure are an applicable and a new method to investigate the effect of loratadine on +Gz tolerance.

In all NIRS variables, as results, no statistically significant differences were found between the subjects on loratadine and placebo (Table 1). In this study including previous our study, HHb increases above its base level during Gz exposure, while cHb and O_2_Hb incurred greater degreases from its base level. Decreases in cerebral cortex oxygen saturation or increases of HHb during +Gz exposure might be the result of arterial and venous left-right shunt and the hydrostatic effect of +Gz. Both O_2_Hb and cHb are sensitive indices in cerebral oxygen saturation increment induced by anti-G maneuver and the anti-G system [23,24]. Kobayashi et al. also reported that the ΔO_2_Hb was negatively correlated with +Gz during aerial combat maneuvering [24]. As noted above, the TOI is useful to evaluate the effect of anti-G protection system [19]. In this study, ΔO_2_Hb/ΔcHb and/or ΔTOI that we observed with great changes in NIRS measurements were considered the quantitative parameter such as +Gz acceleration tolerance. Unfortunately, our sample size was limited (i.e., nine subjects). However, we believe this study represents the first use of a quantitative parameter such as ΔO_2_Hb/ΔcHb and/or ΔTOI to assess the effects of loratadine on acceleration tolerance. NIRS represents a major break-through in research and development.

## Conclusion

No significant differences were observed in +Gz tolerance (+Gz level, duration time and NIRS variables) between subjects taking loratadine and placebo. Our results demonstrate that loratadine has no detectable effect on +Gz tolerance by using a new NIRS method with ΔO_2_Hb/ΔcHb and/or ΔTOI and the traditional method with +Gz level and duration time. This study represents the first use of a quantitative parameter such as ΔO_2_Hb/ΔcHb and/or TOI to assess the effects of a drug on acceleration tolerance.

## Competing interests

The author(s) declare that they have no competing interests.

## Authors' contributions

AO participated in the sequence alignment and draft the manuscript. AZ participated in the design of the study and performed the statistical analysis. YM conceived of the study and in its design and coordination and helped to draft the manuscript. All authors read and approved the final manuscript.
